# Cubic Octa-Carbon: Quantum-Chemical Design of Molecular Structure and Potential Way of Its Synthesis from Cubane

**DOI:** 10.3390/ijms222112067

**Published:** 2021-11-08

**Authors:** Denis V. Chachkov, Oleg V. Mikhailov

**Affiliations:** 1 Kazan Department of Joint Supercomputer Center of Russian Academy of Sciences–Branch of Federal Scientific Center “Scientific Research Institute for System Analysis of the RAS”, Lobachevskii Street 2/31, 420111 Kazan, Russia; de2005c@gmail.com; 2 Analytical Chemistry, Certification and Quality Management Department, Kazan National Research Technological University, K. Marx Street 68, 420015 Kazan, Russia

**Keywords:** octa-carbon, cubane, CCSD method, DFT method

## Abstract

Quantum-chemical calculation of most important parameters of molecular and electronic structures of octa-carbon C_8_ having cubic form (bond lengths, bond and torsion angles) using CCSD(T)/QZVP and DFT B3PW91/QZVP methods, has been carried out. NBO analysis data and HOMO/LUMO images for this compound are presented, too. Good agreement was found between the structural data obtained using the above two quantum-chemical methods and, also, with corresponding experimental data. Also, the standard thermodynamic parameters of formation of cubic C_8_ considered here, and namely standard enthalpy Δ_f_*H^0^*(298K), entropy S_f_^0^(298K) and Gibbs’ energy Δ_f_*G^0^*(298K) of formation for this compound were calculated. By using this data, a theoretically possible variant of the synthesis of this compound by dehydrogenation of cubane C_8_H_8_ is considered, and the thermodynamic characteristics of each of the four stages of this process have been calculated. It is noted that each of the four stages of this process is characterized by a very high (about 500 kJ/mol) enthalpy of activation, as a result of that, for their realization within a sufficiently short time, the use of appropriate catalysts is necessary.

## 1. Introduction

Carbon, alone, among all chemical elements is known to be the only one whose atoms’ number of valence orbitals, number of valence electrons and maximum possible coordination numbers of each coincide with each other. Due to this unique circumstance, it can form long homochain structures containing many hundreds and thousands of atoms. For this element, in addition to the allotropic modifications well and long known to all science—graphite and diamond—a very significant number of other varieties are known, such as carbyne, glassy carbon, various shapes of nanotubes, nanofibers, astralenes, numerous fullerenes (containing, in a molecule, between 60 and several hundred carbon atoms) and amorphous carbon [1,2,3,4,5,6,7,8,9,10]. At the beginning of the twenty-first century another unique modification of this chemical element was obtained—graphene, the structure of which can be considered as one plane of layered graphite, separated from its bulk crystal [1,11,12,13,14,15,16,17]. All these modifications of carbon contain a very significant (at least several dozen) number of atoms in their structural units; nevertheless, according to the data given in [1], there is quite a lot of information in the literature on allotropic modifications of carbon containing a small (less than 10) number of atoms. One of the most interesting modifications of this type is octa-carbon C_8_, for the molecular structure of which the cubic form seems to be the most probable. This substance can serve as a very useful precursor for the organic synthesis of a number of cubane derivatives, for example, polycyclic framework compounds containing articulated cubane fragments; seems to be very promising for use in a number of branches of technology, in particular, as “building blocks” for new nanodevices, which, in turn, can be implemented to create digital memory systems; the possibility of using it in the creation of catalysts for various physicochemical processes is not excluded. The synthesis of such a compound, however, has not yet been experimentally carried out; though there are many works devoted to the theoretical calculation of its molecular and/or electronic structure, carried out mainly using DFT, HF, or even semiempirical methods (see, in particular, [18,19,20,21,22,23,24,25,26,27,28,29,30,31,32]). In this regard, the purpose of this work is to perform a quantum-chemical calculation of the molecular structure of this compound by a more rigorous ab initio quantum-chemical method, namely CCSD (T)/QZVP, as well as to theoretically consider the possibility of its synthesis based on a chemical compound that also has cubic structure, namely, cubane C_8_H_8_.

## 2. Calculation Method

The quantum-chemical calculation of molecular structures of octa-carbon molecule having C_8_ composition was carried out using the CCSD(T)/QZVP method, combining the common QZVP extended quadruple zeta split-valence basis set [33,34] and the coupled cluster method, using both single and double substitutions, including triple excitation non-iterative CCSD(T) [35,36,37,38]. The given method is one of the most accurate and reliable quantum-chemical methods for calculating the molecular structures of various chemical compounds (in particular, *p*-elements) and considers electron correlation very well. Along with this, for comparison, we also calculated the parameters of the molecular structure of C_8_ using the much less time-consuming DFT method, namely DFT B3PW91/QZVP level, which, combined with QZVP and B3PW91 functionality [39,40] and according to data in [41], has minimal values of so-called “normal error” in comparison with other variants of the DFT method. Calculations were performed with the Gaussian09 program package [42]. To visualize the results of our calculations, we used the ChemCraft software, Version 1.8.

**Scheme 1:**
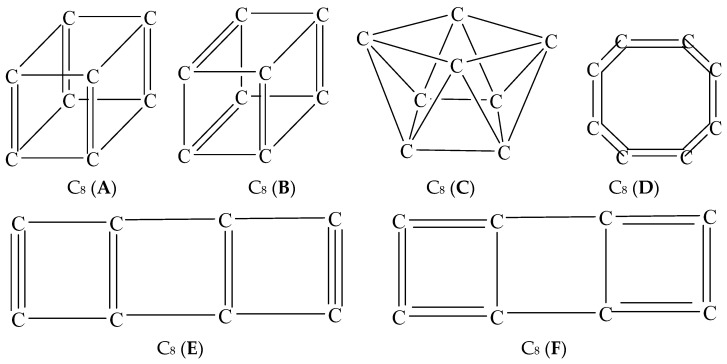
Potential molecular structures of octa-carbon: C_8_ (**A**) and C_8_ (**B**)—cubes, C_8_ (**C**)—square antiprism, C_8_ (**D**)—octagon, C_8_ (**E**) and C_8_ (**F**)—a combination of three rectangles with four common vertices.

The initial structures of the C_8_ molecules for carrying out quantum-chemical calculations are shown in Scheme 1:

The choice of these six structures was determined by the next two factors: firstly, of the valence possibilities of the carbon atom (which can bind with one, two, three, or four neighboring atoms by means of three chemical bonds, according to the exchange mechanism of chemical bond formation); secondly, by these structures’ having the greatest typicality as compared with other structures of eight atoms. That is why, in particular, the hexagonal bipyramid, the dodecahedron and plane bicyclic structure containing articulated six-numbered and four-numbered rings, were not included in the number of initial structures because, here, as well as in other permissible eight-atomic structures, each carbon atom’s being bound to neighboring carbon atoms by exactly four chemical bonds is impossible to achieve. The correspondence of the found stationary points to energy minima was proved, in all cases, by calculating the second derivatives of the energies with respect to atoms’ coordinates; all equilibrium structures corresponding to the minima on the potential energy surfaces have only positive frequencies.

Calculations of the structural parameters of the intermediates and transition states of the theoretically possible reaction for the production of C_8_, and namely, the reaction of the stepwise dehydrogenation of cubane C_8_H_8_, were carried out using the B3PW91/QZVP method, since the use of the coupled-cluster method, CCSD(T)/QZVP, to solve this problem would require very large time and energy costs, far beyond our technical capabilities. In all cases, frequencies of normal vibrations were calculated; in the case of transient states, the first frequency was imaginary and corresponded to the motion of the atoms involved in the reaction. To confirm the correspondence of the found transition states to the studied reaction from each transition state, a descent towards the reagents and reaction products was carried out (IRC procedure in Gaussian09). Of note is that, at the second stage of the dehydrogenation process (i.e., when the second hydrogen molecule is split off), the reaction could already only proceed at those carbon atoms that are chemically bonded to those in C_8_H_6_ molecules deprived of hydrogen atoms. The transition state of the dehydrogenation reaction, in the formation of which hydrogen molecules would be split off from those carbon atoms that were not linked by the above-mentioned chemical bonds, was not found during the calculations.

The values of the standard thermodynamic characteristics of the carbon-containing compounds under study were calculated using the G4 method described, in detail, in [43]. All quantum-chemical calculations were carried out in the Joint Supercomputer Center of the Russian Academy of Sciences-Branch of Federal Scientific Center ”Scientific Research Institute for System Analysis of the RAS” (http://www.jscc.ru).

## 3. Results and Discussion

**Figure 1:**
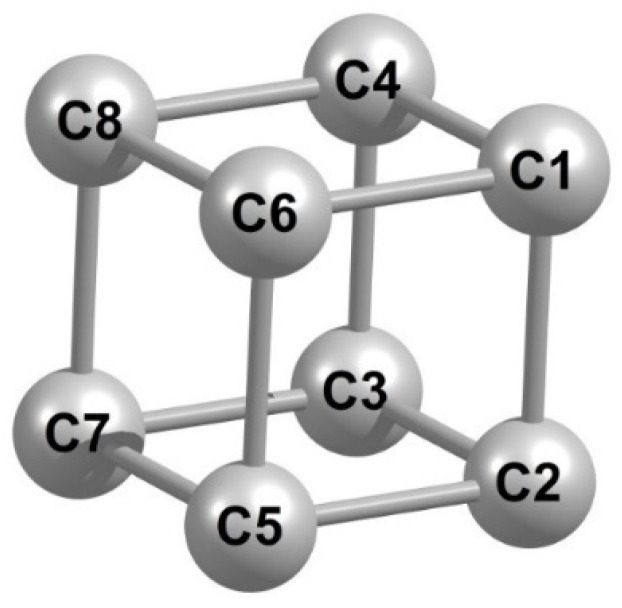
Molecular structure of theoretically possible polymorphic modification of elemental carbon C_8_ according to data of quantum-chemical calculation with using CCSD(T)/QZVP method.

According to the results of our calculations, the most stable of these six structures of octa-atomic carbon molecule mentioned above, is C_8_ (A), in the form of the cube. The image of the molecular structure of this compound calculated by CCSD(T)/QZVP method is shown in Figure 1.

The geometric parameters of this molecular structure calculated with the given methods (bond lengths, bond angles and some torsion angles) are presented in Table 1. As can be seen from these data, quantitatively, the values of similar parameters are very close to each other (the difference between these two structures’ bond lengths between their carbon atoms is no more than 2 pm, and, between their bond and torsion (dihedral) angles, no more than 0.4°). However, there is a very clear qualitative difference between the CCSD(T)/QZVP and DFT B3PW91/QZVP structures: in the former, the bond lengths and angles between carbon atoms are generally not the same (although some of them coincide), whereas, in the latter, they are the same (Table 1). The reasons for this qualitative difference between the results of calculations by the CCSD(T)/QZVP and DFT B3PW91/QZVP methods are most likely associated with the Jahn–Teller effect, and namely, the instability of a highly symmetric structure containing degenerate states of electrons to deformations that lower its symmetry. The point symmetry group *D_4h_* was obtained during the calculation by the DFT B3PW91/QZVP method; in the course of the calculations using the CCSD(T)/QZVP method we obtained the point symmetry group *C_1_*, i.e., a lack of symmetry. Note, in this connection, that within the CCSD(T)/QZVP molecular structure itself the maximum difference between the lengths of carbon–carbon bonds is 0.5 pm, between the bond angles is 0.7°, and between torsion angles is 0.4°; compared with the values of these parameters themselves, these differences look very insignificant.

**Figure 2:**
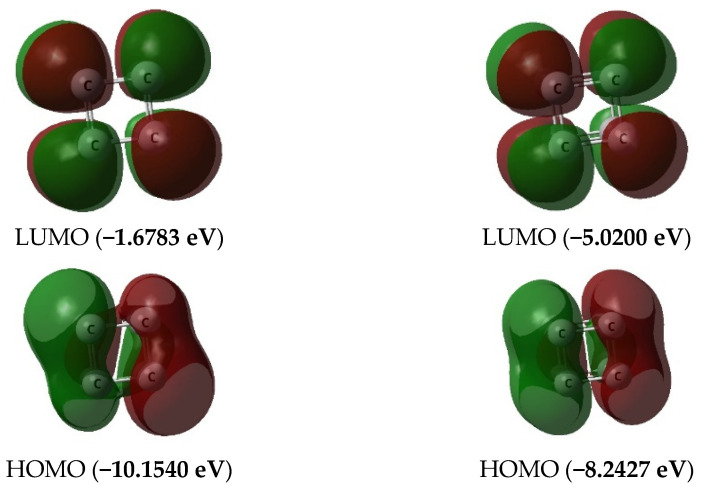
The images of the highest occupied (HOMO) and lowest vacant (LUMO) molecular orbitals obtained by CCSD(T)/QZVP (**left**) and DFT B3PW91/QZVP (**right**).

The images of the highest occupied (HOMO) and lowest vacant (LUMO) molecular orbitals obtained using the above quantum chemical methods are presented in Figure 2. As can be seen therein, the forms of the given MOs obtained by these different methods are very similar to each other, wherein, however, the energies of these MOs are very different from each other. Most likely this difference is associated with a well-known feature of the DFT methods, and namely—due to some approximations that simplify the calculation of the electron correlation—the underestimation, in particular, of the difference in the HOMO-LUMO energies. That is why the data of the coupled-cluster method (CCSD), in the given case, should be considered much more reliable.

NBO analysis data of the C_8_ structures within each of these methods are presented in Table 2. As follows from these data, in the case of using the CCSD(T)/QZVP method, a weakly pronounced polarization of carbon atoms occurs, some of which carry an insignificant (about 0.01 electron charge unit (ē)) positive charge, and some of which carry the same slight negative charge. This feature, as was noted above, is also most likely a result of the Jahn–Teller effect. In the DFT B3PW91/QZVP calculations, the charges on each of the eight carbon atoms, with an accuracy of four significant digits after the decimal, are zero (Table 2).

**Figure 3:**
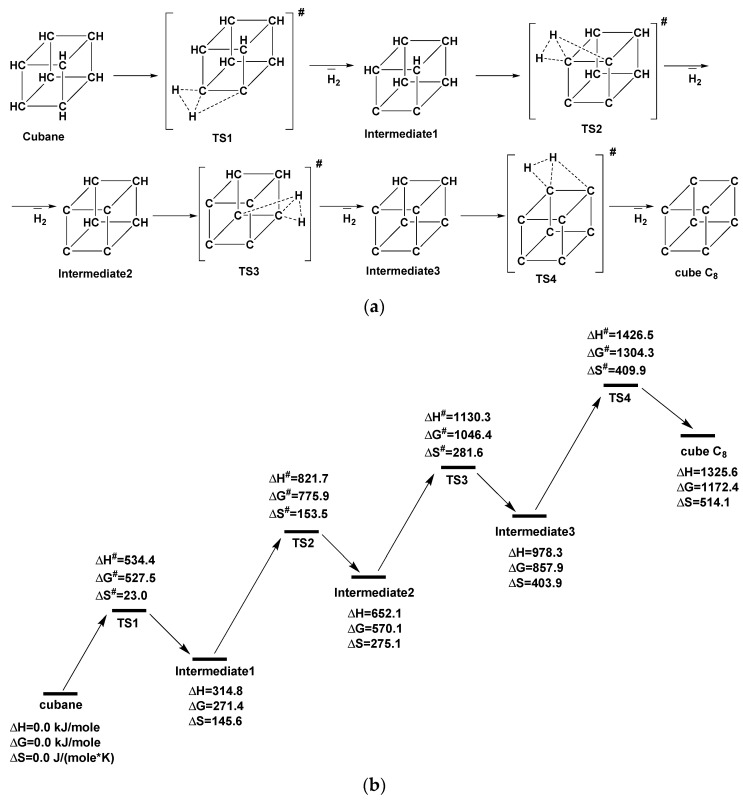
Scheme of the probable dehydrogenation reaction of cubane C_8_H_8_ with the formation of cubic octa-carbon C_8_ (**a**) and the energetic profile of this reaction (**b**) (TS1, TS2, TS3, TS4 are various transient states).

According to our calculations of the standard thermodynamic parameters of the isobaric process (the standard enthalpy of formation Δ_f_*H*^0^(298K), the standard entropy of formation S^0^_f_ (298K), and the standard Gibbs energy of formation Δ_f_*G*^0^(298K)) by the G4 method for the C_8_ molecule, their values are 1871.6 kJ/mol, 348.7 J/mol·K and 1767.6 kJ/mol, respectively. As it can be seen from these data, Δ_f_*H*^0^(298K) and Δ_f_*G*^0^(298K) values are positive (and very significant in modulus), and cannot be obtained for the most stable modification of elemental carbon, graphite, according to the classical canons of thermodynamics. In this connection, it seems advisable to consider the possibility of obtaining cubic octa-carbon in some other way, and one of the most promising methods is the stepwise dehydrogenation of the saturated polycyclic hydrocarbon, cubane C_8_H_8_, which was first obtained more than 50 years ago [44,45] and, by its own the spatial structure, is the compound closest to octa-carbon, according to the general scheme shown in Figure 3.

**Figure 4:**
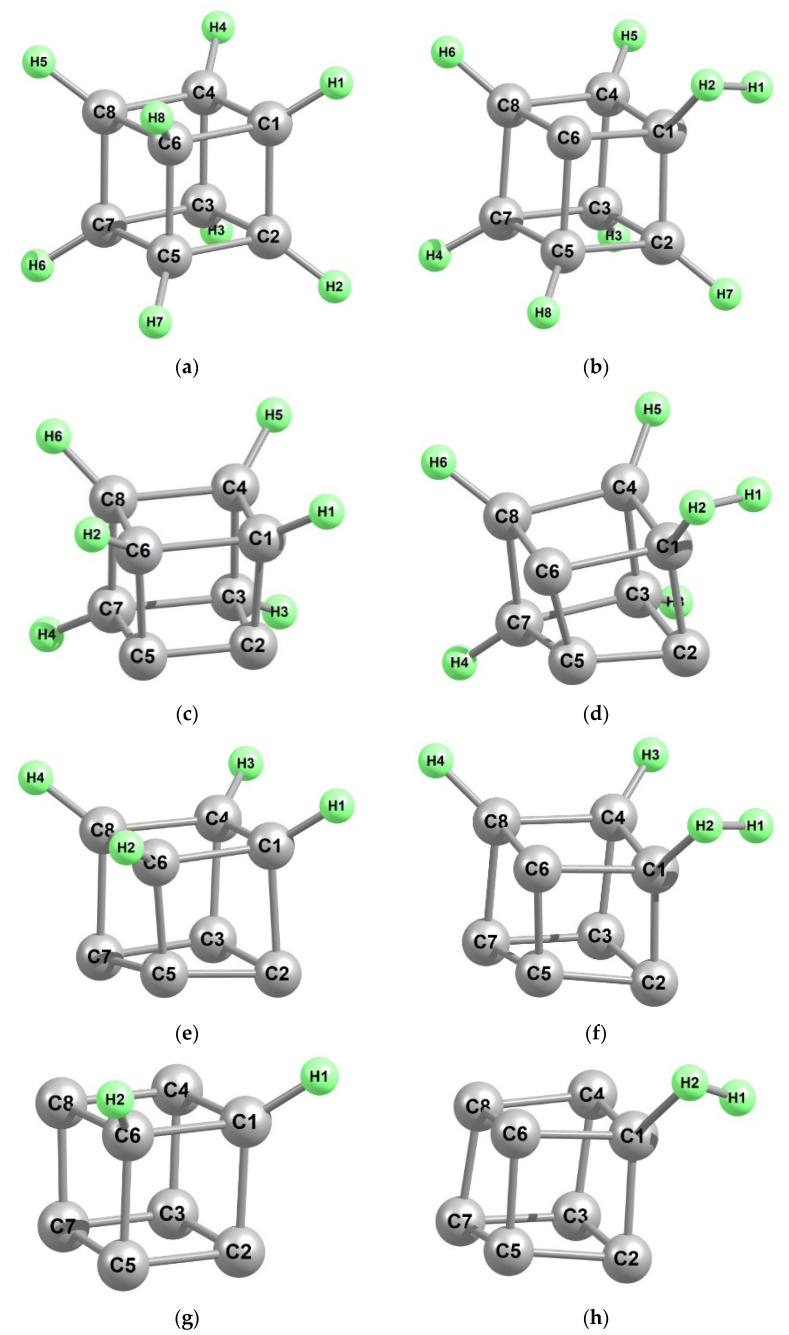
The images of molecular structures of cubane C_8_H_8_ (**a**), intermediates C_8_H_6_ (**c**), C_8_H_4_ (**e**), C_8_H_2_ (**g**) and transient states TS1 (**b**), TS2 (**d**), TS3 (**f**), TS4 (**h**) of reactions of its dehydrogenation.

In accordance with calculation by the DFT B3PW91/QZVP method, the carbon–carbon bond lengths in the cubane molecule are 156.1 pm, the carbon–hydrogen bonds are 108.7 pm, the bond angles are 90.0° and 125.2°, for CCC and CCH, respectively. These values are in very good agreement with the experimental values of these parameters found in [46,47] (157.1 pm, 108.2 pm, 90.0° and 125.0°, respectively). The noted good agreement allows us to assert that, using this method, it is possible to adequately describe the specificity of the dehydrogenation reaction of C_8_H_8_ to C_8_, including the molecular structures of intermediates and transient states TSn (so-called “activated complexes”). The indicated molecular structures are shown in Figure 4, some of their geometric parameters and negative frequency characteristic of the transient states TSn are presented in Table 3 and in Supplementary Materials. As can be seen, the formation of any of the four TSn transient states is accompanied by a rather pronounced deformation of the cubic structure of the initial C_8_H_8_; at the same time, interestingly, in the series TS1–TS2–TS3–TS4, the carbon–carbon bond lengths, as compared to those in cubane, first decrease, then increase, then decrease again and increase again; a similar situation takes place for the carbon–hydrogen bond lengths. The interatomic distances (H1H2), on the contrary, in the same row decrease monotonically (Table 3). In connection with this deformation, the change in the bond angles formed by carbon atoms located in the adjacent vertices of the hexahedron (distorted cube) also looks quite natural, one part of which becomes greater than 90°, while the other part is less than 90°, although the deviation in the values of both these and of other angles from 90° is, as a rule, not more than 5°.

The decomposition reaction of cubane, according to the C_8_H_8_ → C_8_ + 4H_2_ general scheme in both the solid and the gas phase, proceeds with an increase in the total volume of the reaction system (due to the formation of gaseous hydrogen), and therefore, according to the classical canons of thermodynamics, should be accompanied by an increase in the entropy of the reaction system. On the other hand, this reaction is endothermic (according to our calculation, its standard enthalpy is 1325.6 kJ, its standard entropy is 514.1 J/K, and its standard Gibbs’s energy is 1172.4 kJ), so its implementation is provided by the entropy factor. Using the classical Gibbs—Helmholtz equation for the isobaric process Δ_r_*G*^0^(*T*) = Δ_r_*H*^0^(298 K)–*T*Δ_r_*S*^0^(298 K) (where Δ_r_*H*^0^(298 K) and Δ_r_*S*^0^(298 K) are the changes in enthalpy and entropy as a result of a chemical process referred to standard conditions, *T* is the process temperature in K, Δ_r_*G*^0^(*T*) is the dependence of the Gibbs’s free energy on the temperature *T*), it is easy to find that this reaction can proceed at a temperature T ≥ 2578.5 K. A similar situation is observed for individual stages of this reaction with the sequential formation of compounds C_8_H_6_, C_8_H_4_, C_8_H_2_ and C_8_ (see Figure 3).

As can be seen from the data presented in Figure 3b and Table 3, each of the four successive stages of the cubane dehydrogenation reaction is characterized by high values of activation enthalpies and, at the same time, rather low values of activation entropies, as a result of which the energy barriers for the formation of transient states are very significant, wherein the realization of each of these stages is due to the entropy factor, so that all of them can be realized only at sufficiently high temperatures (more than 2000 K). However, even at such high temperatures, the rates of these reactions are likely to be insufficient for their practical implementation within a sufficiently short time, and if so, then in order to increase them, it will be necessary to search for appropriate catalysts for these very reactions.

## 4. Conclusions

As it can be seen from the above, both the used independent quantum-chemical calculation methods—CCSD(T)/QZVP and DFT B3PW91/QZVP—unequivocally indicate the existence of cubic octa-carbon as the most stable of all possible carbon modifications having a C_8_ composition, wherein the quantitative parameters of the molecular structure (bond lengths, bond and torsion (dihedral) angles) of this carbon modification, calculated using these methods, turn out to be very close to each other, though, qualitatively, they are different; according to the data of the first of these methods, as similar parameters are different (though insignificantly), but, according to the second, they are the same (Table 1). Taking into account the fact that, in reality, all carbon atoms in the cubic structure of C_8_ should most likely be equivalent to each other, we can expect that the real structure of cubic octa-carbon C_8_ should be some kind of intermediate variant between the structures obtained as a result of our quantum-chemical calculations using the above independent methods. The assessment of the thermodynamic characteristics of the reaction carried out in this article (from the use of which, in principle, cubic octa-carbon can be obtained, namely, the dehydrogenation of cubane C_8_H_8_) indicates the reality of such a reaction, albeit under rather harsh conditions (i.e., at a high temperature) and with the obligatory use of a catalyst to ensure its flow at a sufficiently high rate. The goal, now, is to confirm all this experimentally.

## Figures and Tables

**Table 1:** Geometric parameters of the molecular structure of C_8_ molecule calculated by the CCSD(T)/QZVP and DFT B3PW91/QZVP calculation methods.

Parameter	Calculated by		Parameter	Calculated by	
	CCSD(T)/QZVP	B3PW91/QZVP		CCSD(T)/QZVP	B3PW91/QZVP
Carbon–Carbon Bond Lengths, *pm*					
(C1C4)	148.3	146.7	(C7C5)	148.0	146.7
(C4C8)	148.0	146.7	(C5C2)	148.6	146.7
(C8C6)	148.2	146.7	(C1C2)	148.3	146.7
(C6C1)	148.1	146.7	(C3C4)	148.3	146.7
(C2C3)	148.3	146.7	(C5C6)	148.5	146.7
(C3C7)	148.5	146.7	(C7C8)	148.5	146.7
Bond Angles, *deg*					
(C1C4C8)	90.4	90.0	(C5C6C1)	90.4	90.0
(C4C8C6)	89.6	90.0	(C6C1C2)	89.8	90.0
(C8C6C1)	90.3	90.0	(C6C5C7)	89.9	90.0
(C6C1C4)	89.7	90.0	(C5C7C8)	90.2	90.0
(C2C5C7)	89.9	90.0	(C7C8C6)	89.8	90.0
(C5C7C3)	90.3	90.0	(C8C6C5)	90.1	90.0
(C1C2C3)	90.2	90.0	(C7C8C4)	89.8	90.0
(C2C3C4)	89.8	90.0	(C8C4C3)	90.4	90.0
(C3C4C1)	90.2	90.0	(C4C3C7)	89.7	90.0
(C6C1C4)	89.7	90.0	(C3C7C8)	90.1	90.0
(C1C2C5)	90.3	90.0	(C7C8C4)	89.8	90.0
(C2C5C6)	89.9	90.0	(C8C4C3)	90.4	90.0
Selected Torsion (Dihedral) Angles, *deg*					
(C1C4C8C6)	–0.2	0.0	(C1C2C7C8)	0.0	0.0
(C1C4C3C2)	0.4	0.0	(C4C6C5C3)	0.2	0.0
(C1C2C5C6)	0.0	0.0	(C1C2C3C7)	–90.1	–90.0
(C2C3C7C5)	–0.2	0.0	(C2C5C6C8)	90.3	90.0
(C5C6C8C7)	–0.4	0.0	(C5C6C8C4)	–90.2	–90.0
(C3C4C8C7)	0.2	0.0	(C7C8C4C1)	–90.0	–90.0

**Table 2:** NBO analysis data for the cubic octa-carbon C_8_ calculated by the CCSD(T)/QZVP and DFT B3PW91/QZVP methods.

Calculation Method	Effective Charge on Carbon Atoms, in Units of Electron Charge (ē)							
	C1	C2	C3	C4	C5	C6	C7	C8
CCSD(T)/QZVP	−0.0104	+0.0099	−0.0098	+0.0102	−0.0097	+0.0102	+0.0099	−0.0103
B3PW91/QZVP	0.0000	0.0000	0.0000	0.0000	0.0000	0.0000	0.0000	0.0000

**Table 3:** Enthalpy of activation (Δ*H*^#^, kJ/mol), Gibbs’s energy of activation (Δ*G*^#^, kJ/mol), entropy of activation (Δ*S*^#^, J/mol·K) under standard conditions, standard enthalpy of reaction (Δ*H*_r_, kJ), the standard Gibbs’s energy of the reaction (Δ*G*_r_, kJ), the standard entropy of the reaction (Δ*S*_r_, J/K), some geometric parameters and negative frequency characteristic of the transient states TSn (n = 1–4, see Figure 3 and Figure 4) of the dehydrogenation process cubane calculated by the DFT B3PW91/QZVP method.

Transition Stage (TSn)	TS1	TS2	TS3	TS4
Δ*H*^# ^ Δ*G*^# ^ Δ*S*^#^	534.4 527.5 23.0	506.9 504.5 8.0	478.2 476.3 6.5	448.2 446.5 5.9
Δ*H*_r_ Δ*G*_r _ Δ*S*_r_	314.8 271.4 145.6	337.3 298.6 129.6	326.2 287.8 128.8	347.3 314.5 110.1
r(H1H2), *pm*	90.9	90.4	85.2	84.3
r(C1H1), *pm*	140.2	134.3	144.4	137.6
r(C1H2), *pm*	125.5	124.4	132.8	134.5
r(C2H2), *pm*	197.2	209.4	218.9	219.2
r(C1C2), *pm*	155.0	160.7	157.7	154.4
∠H1C1H2, *deg*	39.6	40.7	35.5	36.1
∠H2C1C2, *deg*	88.7	93.7	97.4	98.5
ν_1_, cm^−1^	−1569	−1258	−1144	−1022

## Supplementary Materials

The following are available online at https://www.mdpi.com/article/10.3390/ijms222112067/s1.
