# Supplementary material for: Cubic Octa-Carbon: Quantum-Chemical Design of Molecular Structure and Potential Way of Its Synthesis from Cubane
Source: Int J Mol Sci. 2021 Nov 8;22(21):12067. doi: 10.3390/ijms222112067 (PMC8584512; doi:10.3390/ijms222112067)
Supplement: Supplementary file 1 [file ijms-22-12067-s001.zip › ijms-1411097-supplementary.pdf]

Supplementary Materials

The key geometric paraments of cubane C_8H_n molecular structure

**Bond lengths, *pm***			
(C1C2)	156.1	(C4C8)	156.1
(C1C4)	156.1	(C4H4)	108.7
(C1C6)	156.1	(C5C6)	156.1
(C1H1)	108.7	(C5C7)	156.1
(C2C3)	156.1	(C5H7)	108.7
(C2C5)	156.1	(C6C8)	156.1
(C2H2)	108.7	(C6H8)	108.7
(C3C4)	156.1	(C7C8)	156.1
(C3C7)	156.1	(C7H6)	108.7
(C3H3)	108.7	(C8H5)	108.7
**Bond angles, *deg***			
(C2C1C4)	90.0	(C2C5C6)	90.0
(C2C1C6)	90.0	(C2C5C7)	90.0
(C2C1H1)	125.3	(C2C5H7)	125.3
(C4C1C6)	90.0	(C6C5C7)	90.0
(C4C1H1)	125.3	(C6C5H7)	125.3
(C6C1H1)	125.3	(C7C5H7)	125.3
(C1C2C3)	90.0	(C1C6C5)	90.0
(C1C2C5)	90.0	(C1C6C8)	90.0
(C1C2H2)	125.3	(C1C6H8)	125.3
(C3C2C5)	90.0	(C5C6C8)	90.0
(C3C2H2)	125.3	(C5C6H8)	125.3
(C5C2H2)	125.3	(C8C6H8)	125.3
(C2C3C4)	90.0	(C3C7C5)	90.0
(C2C3C7)	90.0	(C3C7C8)	90.0
(C2C3H3)	125.3	(C3C7H6)	125.3
(C4C3C7)	90.0	(C5C7C8)	90.0
(C4C3H3)	125.3	(C5C7H6)	125.3
(C7C3H3)	125.3	(C8C7H6)	125.3
(C1C4C3)	90.0	(C4C8C6)	90.0
(C1C4C8)	90.0	(C4C8C7)	90.0
(C1C4C12)	125.3	(C4C8H5)	125.3
(C3C4C8)	90.0	(C6C8C7)	90.0
(C3C4H4)	125.3	(C6C8H5)	125.3
(C8C4H4)	125.3	(C7C8H5)	125.3
**Selected torsion (dihedral) angles, *deg***			
(C4C1C2C3)	0.0	(C2C3C7C5)	0.0
(C4C1C2C5)	-90.0	(C2C3C7C8)	90.0
(C4C1C2H2)	135.0	(C2C3C7H6)	-135.0
(C6C1C2C3)	90.0	(C4C3C7C5)	-90.0
(C6C1C2C5)	0.0	(C4C3C7C8)	0.0
(C6C1C2H2)	-135.0	(C4C3C7H6)	135.0
(H1C1C2C3)	-135.0	(H3C3C7C5)	135.0
(H1C1C2C5)	135.0	(H3C3C7C8)	-135.0
(H1C1C2H2)	0.0	(H3C3C7H6)	0.0
(C2C1C4C3)	0.0	(C1C4C8C6)	0.0
(C2C1C4C8)	90.0	(C1C4C8C7)	-90.0
(C2C1C4H4)	-135.0	(C1C4C8H5)	135.0
(C6C1C4C3)	-90.0	(C3C4C8C6)	90.0
(C6C1C4C8)	0.0	(C3C4C8C7)	0.0

The images of intermediate C_8H_n (n= 8, 6, 4, 2) molecular structures of the cubane dehydrogenation reaction

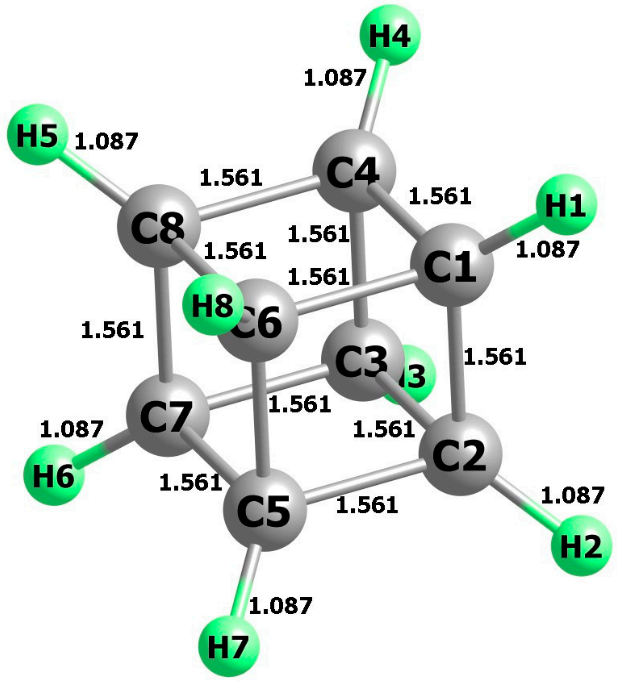

C_8H_8

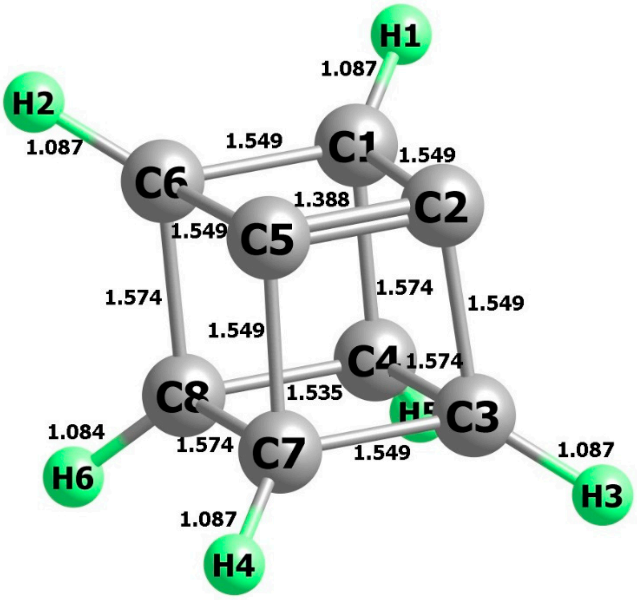

C_8H_6

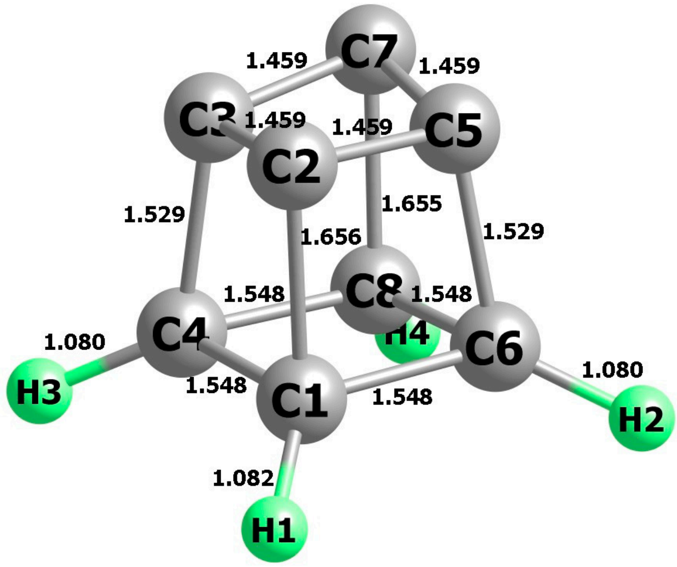

C_8H_4

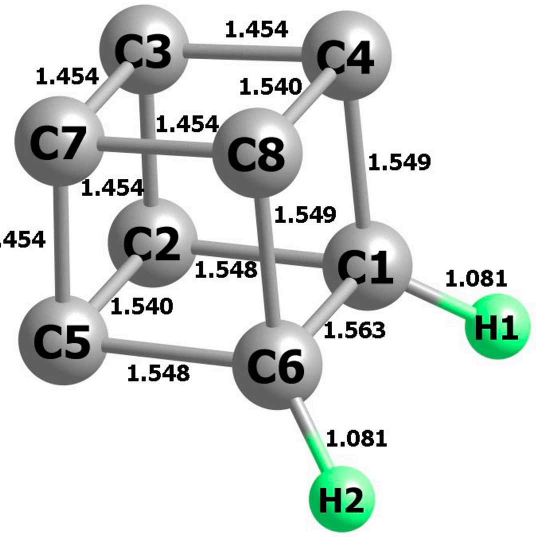

C_8H_2

The images of transient states (TS1, TS2, TS3 and TS4) of the cubane dehydrogenation reaction

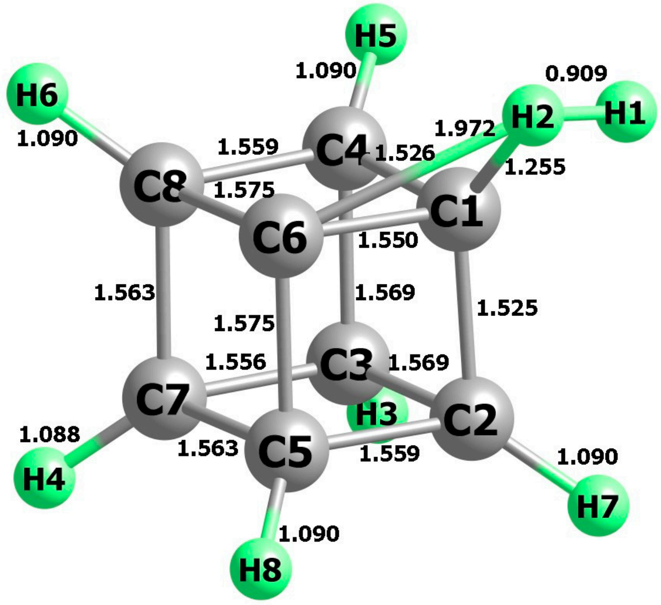

TS1

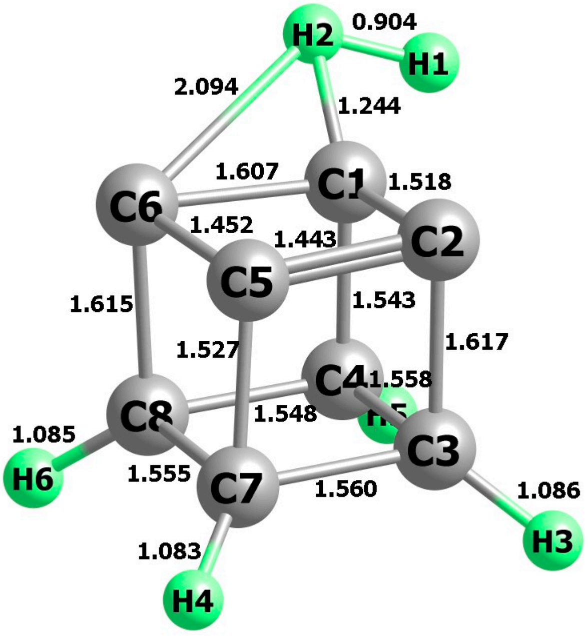

TS2

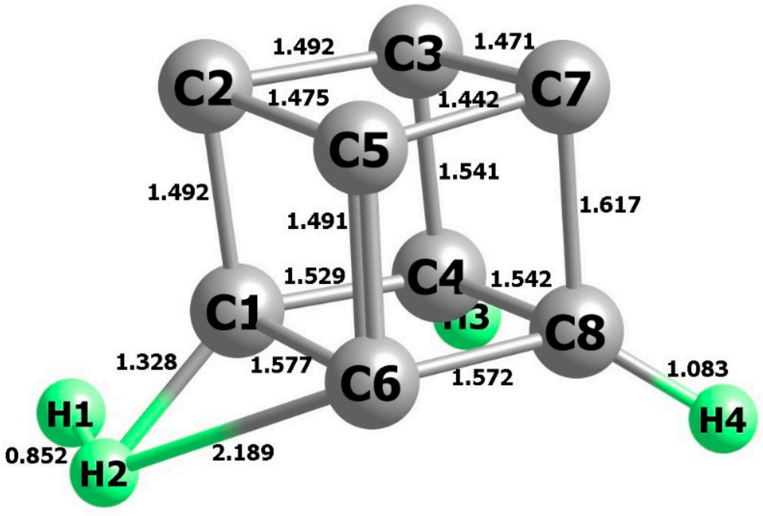

TS3

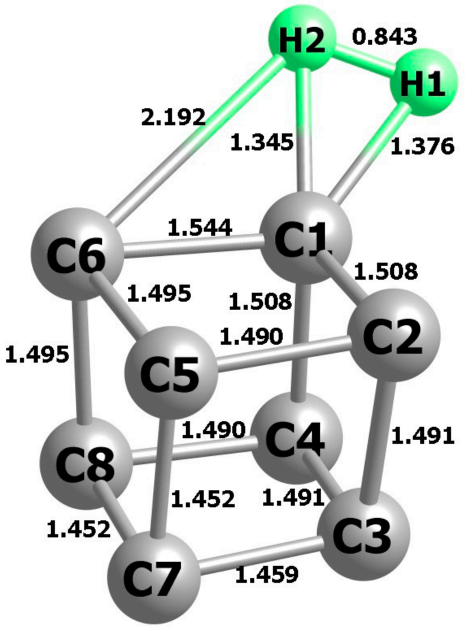

TS4
